# A snake venom group IIA PLA_2_ with immunomodulatory activity induces formation of lipid droplets containing 15-d-PGJ_2_ in macrophages

**DOI:** 10.1038/s41598-017-04498-8

**Published:** 2017-06-22

**Authors:** Karina Cristina Giannotti, Elbio Leiguez, Ana Eduarda Zulim de Carvalho, Neide Galvão Nascimento, Márcio Hideki Matsubara, Consuelo Latorre Fortes-Dias, Vanessa Moreira, Catarina Teixeira

**Affiliations:** 1 Pharmacology Laboratory, Butantan Institute, São Paulo, SP Brazil; 2 Molecular Biology and Bioinformatics Laboratory, Ezequiel Dias Foundation, Belo Horizonte, MG Brazil; 3 Department of Pharmacology, Paulista School of Medicine, Federal University of São Paulo, São Paulo, CEP 04044-020 Brazil

## Abstract

Crotoxin B (CB) is a catalytically active group IIA sPLA_2_ from *Crotalus durissus terrificus* snake venom. In contrast to most GIIA sPLA_2_s, CB exhibits anti-inflammatory effects, including the ability to inhibit leukocyte functions. Lipid droplets (LDs) are lipid-rich organelles associated with inflammation and recognized as a site for the synthesis of inflammatory lipid mediators. Here, the ability of CB to induce formation of LDs and the mechanisms involved in this effect were investigated in isolated macrophages. The profile of CB-induced 15-d-PGJ_2_ (15-Deoxy-Delta-12,14-prostaglandin J_2_) production and involvement of LDs in 15-d-PGJ_2_ biosynthesis were also investigated. Stimulation of murine macrophages with CB induced increased number of LDs and release of 15-d-PGJ_2_. LDs induced by CB were associated to PLIN2 recruitment and expression and required activation of PKC, PI3K, MEK1/2, JNK, iPLA_2_ and PLD. Both 15-d-PGJ_2_ and COX-1 were found in CB-induced LDs indicating that LDs contribute to the inhibitory effects of CB by acting as platform for synthesis of 15-d-PGJ_2_, a pro-resolving lipid mediator. Together, our data indicate that an immunomodulatory GIIA sPLA_2_ can directly induce LD formation and production of a pro-resolving mediator in an inflammatory cell and afford new insights into the roles of LDs in resolution of inflammatory processes.

## Introduction

Phospholipases A_2_ (PLA_2_s) are enzymes that play a key role in a range of cellular processes in physiological and pathological conditions by regulating the release of arachidonic acid (AA), a precursor of distinct classes of lipid mediators such as prostaglandins and leukotrienes, which are key regulators of inflammatory processes^1^. These enzymes have been classified into fifteen groups (I–XV) and subgroups according to nucleotide and amino acid sequence criteria. The groups comprise five distinct types of enzymes: secreted (sPLA_2_), cytosolic (cPLA_2_) and calcium independent (iPLA_2_) PLA_2_s, platelet activating factor (PAF) acetylhydrolases and lysosomal PLA_2_s^2^. Group IIA includes mammalian inflammatory sPLA_2_s and phospolipases present in viperid snake venoms^3^. Crotoxin B (CB), a group IIA sPLA_2_ isolated from *Crotalus durissus terrificus (Cdt)* snake venom, is one of the two subunits that form crotoxin, the major component of *Cdt* venom. In contrast to most group IIA sPLA_2_s, CB per se displays immunomodulatory effects^4, 5^. It is able to inhibit macrophage spreading and phagocytic activities, both of which are associated with increased levels of lipoxin A_4_ (LXA_4_), an important arachidonic acid-derived lipid mediator shown to be active in the resolution phase of inflammation^6^. CB also induces the biosynthesis and release of prostaglandin E_2_ (PGE_2_) and D_2_ (PGD_2_) via activation of the catalytic activity of cyclooxygenase-1 (COX-1)^7^. Although PGE_2_ is known to dilate blood vessels, potentiating edema formation, its immunosuppressive activities, including inhibition of the phagocytic functions and microbicidal activity of macrophages, have been well demonstrated^8–10^. Similarly, while PGD_2_ is an important mediator of the acute inflammatory response, its hydrolysis product, 15-deoxy^12–14^ PGJ_2_ (15-d-PGJ_2_), is a major mediator during the resolution phase of inflammation, regulating the switch from acute inflammation to active inflammatory resolution^11, 12^. Despite the importance of 15-d-PGJ_2_ as a pro-resolving mediator, there is currently no information in the literature about its production by innate immune cells stimulated by group IIA PLA_2_s, including CB.

Under inflammatory and infectious conditions, prostaglandins and other lipid mediators can be synthesized by cytoplasmic organelles known as lipid droplets (LDs), which are lipid-rich structures composed of a neutral lipid core surrounded by a monolayer of phospholipids^13^. These organelles compartmentalize key enzymes involved in the synthesis of lipid mediators, such as COX-1, COX-2 and 5-lypoxigenase, as well as proteins related to membrane trafficking, cell signaling, such as kinases, and structural proteins, such as perilipin 2 (PLIN2)^14–17^, an important protein involved in LD assembly, adipocyte differentiation and foam cell formation^18–20^. LDs have been shown to play central roles in the heightened production of lipid mediators, which in turn drive the evolution of the inflammatory response^21, 22^. Indeed, increased numbers of LDs have been observed in leukocytes in a number of clinical and experimentally induced inflammatory diseases^23, 24^.

Recently, the inflammatory group IIA sPLA_2_s have emerged as critical regulators of LD formation, as they provide free fatty acids from membrane phospholipids that are crucial for this process, directly regulating assembly of these organelles^25^. In light of this and the fact that CB, a group IIA sPLA_2_, is able to release arachidonic acid^7^, a major fatty acid involved in both LD formation and lipid-mediator production^26, 27^ during inflammatory processes, we decided to investigate the ability of CB to induce LD formation and the mechanisms involved in LD formation, as well as 15-d-PGJ_2_ production, in macrophages. We also investigated the involvement of LDs in CB-induced 15-d-PGJ_2_ biosynthesis as well as the involvement of COX-1, COX-2, cPLA_2_, iPLA_2_ and the signaling pathway proteins PI3K, PKC, MEK1/2 and JNK in LD formation.

## Materials and Methods

### Reagents and chemicals

Hema-3 stain was purchased from Biochemical Sciences Inc. (Swedesboro, NJ, USA). Brewer thioglycolate medium was purchased from Difco, Surrey, UK and RPMI 1640 from Thermo Scientific, Waltham, MA, USA. MTT and L-glutamine were purchased from USB Corporation (Cleveland, OH, USA). H7, SB202190, PD98059, JNK inhibitor II and Pyr-2 were purchased from Calbiochem-Novabiochem Corp. (La Jolla, CA, USA), and racemic BEL, FKGK11, CAL-101, ERK inhibitor, HQL-79 and Valeryl Salicylate from Cayman Chemical (Ann Arbor, MI, USA). SB203580, GF109203X and SP600125X were purchased from Sigma-Aldrich Corporation (St. Louis, MO, USA). U0126, phospho and total-PKC, -PI3K, -MEK1/2 and -JNK were obtained from Cell Signaling Technology Inc. (Danvers, MA, USA). SC-560 was a kindly supplied by Dr. Alexandre A. Steiner – Dept. of Immunology, ICB, Sao Paulo University, Brazil. Horseradish peroxidase conjugated goat anti-rabbit IgG antibody, mouse monoclonal anti-β-actin antibody and Nile Red were purchased from Sigma-Aldrich Corporation (St. Louis, MO, USA). Guinea pig polyclonal anti-mouse PLIN2 antibody and FITC-conjugated donkey anti-guinea pig antibody were purchased from Research Diagnostics Inc. (Flanders, NJ, USA). Secondary anti-mouse and anti-guinea pig horseradish peroxidase conjugated antibodies and nitrocellulose membrane were purchased from GE Healthcare (Buckinghamshire, UK). Gentamicin was purchased from Schering-Plough Corporation (Kenilworth, NJ, USA). DMSO and BSA were purchased from Amresco Inc. (Cleveland, OH, USA). RPMI-1640, thiocarbohydrazide, OsO_4_, N-ethyl-N′-(3-dimethylaminopropyl) carbodiimide hydrochloride (EDAC) and RIPA Buffer (lysis buffer) were purchased from Sigma-Aldrich (Swedesboro, NJ, USA). All the salts used were purchased from Merck KgaA (Darmstadt, Germany). Paraformaldehyde was purchased from Electron Microscopy Sciences (Hatfield, PA, USA). Alexa Fluor 488 goat anti-mouse IgG was purchased from Life Technologies Corporation (Carlsbad, CA, USA) and prostaglandin E_2_, prostaglandin J_2_ and cyclooxigenase-1 monoclonal antibodies from Cayman Chemical (Ann Arbor, MI, USA). Fluoromount-G was purchased from Molecular Probes (Eugene, OR, USA). Donkey serum was purchased from Jackson ImmunoResearch Laboratories, Inc. (West Grove, PA, USA), Triton-X from Union Carbide Corporation (Houston, TX, USA).

### Animals

Male Swiss mice (18–20 g) from the Butantan Institute (Sao Paulo, Brazil) were used. The animals were housed in a temperature-controlled room (22–24 °C) with a 12 h light-dark cycle and received fresh water and food *ad libitum* until they were used. The study was approved by the Butantan Institute Animal Experimentation Ethics Committee (reference no. 846/11) in accordance with the procedures laid down by the Universities Federation for Animal Welfare.

### Phospholipase A_2_

CB-sPLA_2_ was obtained from crotoxin (CTX) isolated from *Cdt* snake venom by reversed-phase chromatography^27, 28^, using a 201SP54 column (Grace Vydac, CA, USA). Briefly, 1 mg of CTX was loaded onto the column, and fractions of 0.2 mL were eluted with a linear gradient of 0.1% trifluoroacetic acid (TFA) in H_2_O and 0.1% TFA in acetonitrile. CTX was isolated from *Cdt* venom by conventional gel filtration on Sephadex 75^29^. CB identity was checked by SDS-polyacrylamide gel electrophoresis under non-reducing conditions and by matrix-assisted laser desorption ionization time-of-flight mass spectrometry in an AutoFlex III MALDI-TOF-TOF instrument (Bruker Daltonics, Bremen, Germany). The PLA_2_ activity of CB was assayed using the classical egg yolk clearing method^30^. The absence of endotoxin contamination in the CB preparation was demonstrated by the quantitative *Limulus* Amebocyte Lysate (LAL) test^31^, which revealed undetectable levels of endotoxin (<0.125 EU/mL).

### Macrophage harvesting

Elicited macrophages were harvested four days after i.p. injection of 1 mL of 3% Brewer thioglycolate medium. The animals were killed under CO_2_ atmosphere, and the cells were harvested by washing the peritoneal cavities with 3 mL of PBS, pH 7.2, containing 10 IU/mL heparin. Aliquots of the washes were used for total cell counts in a Neubauer chamber after dilution (1:20, v/v) in Turk’s solution (0.2% crystal violet dissolved in 30% acetic acid). Differential cell counts were performed on smears stained with Hema-3 and examined under light microscopy. More than 95% of the cell population consisted of macrophages according to conventional morphological criteria. The remaining peritoneal wash was centrifuged at 500 *g* for 6 min (4 °C), and the cell pellets were used for subsequent studies after suitable dilutions.

### Cytotoxicity assay

Cytotoxicity of CB and all the pharmacological agents used toward thioglycolate-elicited macrophages was evaluated using a tetrazolium-based assay (MTT). In brief, 2 × 10^5^ macrophages/well in RPMI-1640 medium supplemented with 40 µg/mL gentamicin sulfate and 2 mM L-glutamine were plated in 96-well plates and incubated with 100 μL of selected concentrations of CB (0.1–0.8 μM) diluted in medium or with the same volume of medium alone (control) for 1, 3, 6, 12 or 24 h at 37 °C in a humidified atmosphere of 5% CO_2_. MTT (5 mg/mL) was dissolved in PBS and filtered to sterilize it and remove a small amount of insoluble residue present in some batches. Stock MTT solution (10% in culture medium) was added to all wells in each assay, and the plates were incubated at 37 °C for 3 h. Next, a volume of 100 μL of DMSO was added to the wells and mixed thoroughly at room temperature for 30 min. Absorbance at 540 nm was then recorded in a microtiter plate reader. Results were expressed as percentage of viable cells, and the control cells (incubated with medium alone) were considered 100% viable.

### Macrophage culture and stimulation

Macrophages were plated on glass coverslips in 24-well plates at a density of 2 × 10^5^ cells/coverslip and allowed to attach for 30 min at 37 °C under a 5% CO_2_ atmosphere. Non-adherent cells were removed by washing with PBS. Cell monolayers were cultured for 1 h in RPMI-1640 supplemented with 40 μg/mL gentamicin sulfate and 2 mM L-glutamine at 37 °C and 5% CO_2_ and were then challenged with selected concentrations of CB (0.1–0.8 μM) or culture medium alone (control). Where appropriate, the following inhibitors were used: 6 μM H7 or 1 μM GF109203X, both inhibitors of PKC (protein kinase C); 1 μM LY294002 or 1 μM CAL-101, both inhibitors of PI3K (phosphoinositide 3-kinase); 1 μM U73122, an inhibitor of PLC (phospholipase C); 1 μM FIPI, an inhibitor of PLD (phospholipase D); 2 μM JNK inhibitor II or 10 μM SP600125, both inhibitors of JNK (c-Jun N-terminal kinase); 25 μM U0126, an inhibitor of MEK1/2; 5 μM SB202190 or 16 μM SB203580, inhibitors of p38^MAPK^ (mitogen-activated protein kinase); 25 μM PD98059 or 5 μM ERK inhibitor, both inhibitors of ERK1/2; 1 μM Pyr-2 (Pyrrolidine-2), an inhibitor of cPLA_2_; 2 μM BEL (bromoenol lactone) or 1μM FKGK11, both inhibitors of iPLA_2_, 30 μM HQL-79, an inhibitor of PGD synthase and 450 μM valeryl salicylate or 0.5 μM SC-560, both inhibitors of COX-1. All the stock solutions were prepared in DMSO and stored at −20 °C. Aliquots were diluted in RPMI-1640 immediately before use to give the required concentration. The final DMSO concentration was always lower than 1% and had no effect on the number of LDs. Pharmacological inhibitors were added 30 or 60 min before stimulation of macrophages with CB or culture medium (control) at concentrations previously tested^32–44^. Cells treated with the inhibitors were analyzed for viability with the MTT assay. No significant changes in cell viability were registered with any of the above agents or the vehicle at the concentrations used (data not shown).

### Lipid droplet staining and quantification

Analysis of LD numbers was performed in osmium-stained cells. In brief, macrophages (2 × 10^5^ cells) adhered to glass coverslips were fixed in 4% paraformaldehyde (PFA) in 0.1 M phosphate buffer, pH 7.2, for 15 min and stained with OsO_4_ (osmium tetroxide). The coverslips were then rinsed in 0.1 M phosphate buffer, stained in 1% OsO_4_ (30 min), rinsed in deionized H_2_O, immersed in 1.0% thiocarbohydrazide (5 min), rinsed again in 0.1 M phosphate buffer, re-stained with 1% OsO_4_ (3 min), rinsed with H_2_O and then dried and mounted. The morphology of the fixed cells was examined, and round osmiophilic structures were identified as LDs, which were then counted under phase-contrast microscopy using a 100x objective lens in 50 consecutively scanned macrophages in each coverslip. For assays with fluorescent-labeled LDs, macrophages (2 × 10^5^ cells) adhered to glass coverslips were incubated with Nile Red staining solution freshly prepared in 0.1 M phosphate buffer (10 μg/mL) for 20 min at room temperature and washed with phosphate buffer. After several washes the coverslips were mounted with Fluoromount-G and examined under a confocal microscope (Zeiss LSM 510 Meta confocal microscope).

### Immunocytochemistry analysis

Detection of PLIN2 in CB-stimulated macrophages was performed by PLIN2 immunostaining. Cells were fixed in 2% PFA, permeabilized with 0.2% Triton-X 100 in 0.1 M phosphate buffer and blocked with 0.5% normal donkey serum in 0.1 M phosphate buffer for 90 min. After PBS washes, macrophages were incubated for 1 h with guinea pig polyclonal anti-mouse PLIN2 (1:2000) diluted in 0.1 M phosphate buffer with 0.2% Triton-X 100. After three washes with PBS (10 min each), the preparations were incubated with secondary FITC-conjugated donkey anti-guinea pig antibody (1:500) in the dark for 1 h. After the washes, the slides were mounted with Fluoromount-G and examined under a confocal laser scanning microscope (Zeiss LSM 510 Meta). For analysis of COX-1and 15-d-PGJ_2_ immunostaining, the cells were fixed and permeabilized in 1% EDAC^45^ in calcium- and magnesium-free Hank balanced salt solution (HBSS^−/−^). The macrophages were blocked with 0.5% normal donkey serum in 0.1 M phosphate buffer for 60 min and then washed with HBSS^−/−^ and incubated for 1 h with monoclonal antibodies against COX-1 or 15-d-PGJ_2_ (1:100). After further washes, the cells were incubated with goat anti-mouse Alexa Fluor 488 or goat anti-rabbit Alexa Fluor 488 (1:250) and Nile Red solution (1:250) for 1 h. The coverslips were then washed three times and mounted with Fluoromount-G and examined under a confocal laser scanning microscope (Zeiss LSM 510 Meta).

### Western blotting

For PLIN2 detection, whole cells extracts obtained by lysing the pellets with 100 μL of sample buffer (0.5 M Tris–HCl, pH 6.8, 20% SDS, 1% glycerol, 1 M β-mercaptoethanol, 0.1% bromophenol blue) and boiling them for 10 min were used. Samples were resolved by SDS polyacrylamide gel electrophoresis (SDS-PAGE) on a 10% bis-acrylamide gel overlaid with a 5% stacking gel. Proteins were then transferred to a nitrocellulose membrane (GE Healthcare, Buckinghamshire, UK) using a Mini Trans-Blot® (Bio-Rad Laboratories, Richmond, CA, USA). The membranes were blocked for 1 h with 5% nonfat dry milk in TTBS (20 mM Tris, 100 mM NaCl and 0.5% Tween 20) and incubated with a primary antibody against PLIN2 (Abcam) overnight at 4 °C or with β-actin (Sigma) for 1 h at room temperature. They were then washed and incubated with the appropriate horseradish peroxidase conjugated anti-rabbit IgG secondary antibody (1:1000 dilution, 1 h, room temperature). To analyze phosphorylated forms of PI3K, MEK1/2, ERK1/2 and JNK, the cells were lysed using RIPA^®^ (Sigma Aldrich) with a protease inhibitor cocktail and phosphatase inhibitors (sodium fluorate 10 mM and sodium orthovanadate 1 mM) and then dissolved (1:5 v/v) in the sample buffer and boiled for 10 min. After the proteins had been separated by SDS-PAGE (12%) and transferred to a nitrocellulose membrane, as described above, the phosphorylated and total non-phosphorylated proteins were detected using polyclonal antibodies to PI3K, MEK1/2, ERK1/2 and JNK (1:1000) and total kinases (1:650) overnight at 4 °C, followed by incubation with horseradish peroxidase-conjugated anti-rabbit IgG (1:1000, 1 h, room temperature). Detection was by the enhanced chemiluminescence (ECL) method according to the manufacturer’s instructions (GE Healthcare, Buckinghamshire, UK). Band densities were quantified with a GS 800 Densitometer (Bio-Rad Laboratories, Richmond, CA) using Molecular Analyst^®^ image analysis software (Bio-Rad Laboratories, Richmond, CA).

### Quantification of 15-d-PGJ_2_ concentrations

15-d-PGJ_2_ (15-deoxy^12–14^ PGJ_2_) concentrations were determined by a specific enzyme immunoassay using a commercial kit (Enzo life Sciences, Inc. New York, USA) as per manufacturer’s protocol.

### Statistical Analysis

Data are expressed as the mean ± standard error of mean (SEM) of at least three independent experiments. Multiple comparisons among groups were performed with one-way ANOVA followed by Tukey’s test. Probabilities of less than 5% (*p* < 0.05) were considered statistically significant.

## Results

### CB induces LDs formation in macrophages

**Figure 1:**
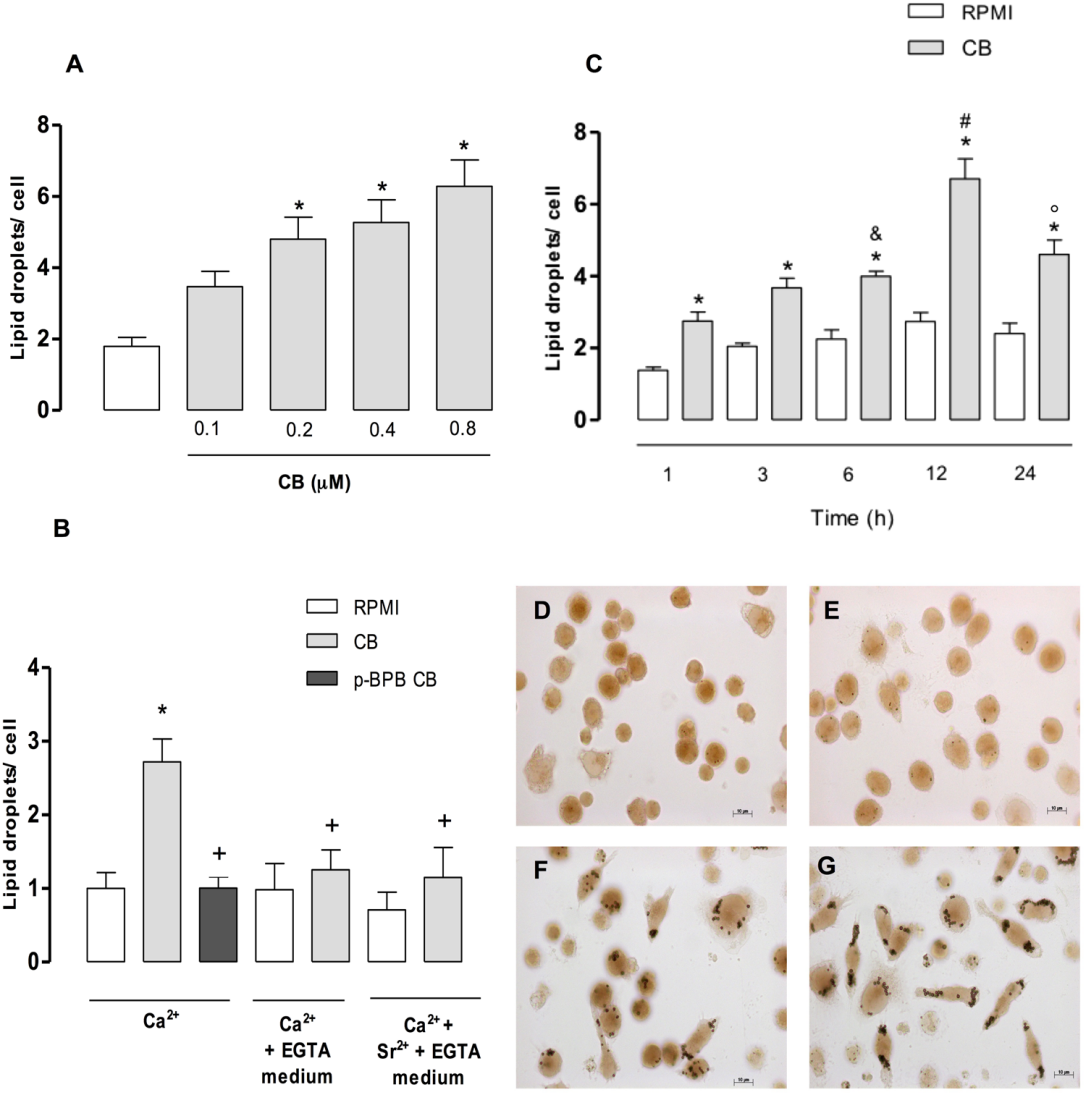
CB induces LD formation in peritoneal macrophages. (**A**) Effect of CB on LD formation in macrophages incubated with selected concentrations of CB or with RPMI (control) for 1 h. (**B**) Effect of catalytically active CB on LD formation in peritoneal macrophages incubated with one of the following for 1 h: (i) CB (0.4 μM) in a medium containing EGTA (200 μM) with or without Ca^2+^; (ii) CB (0.4 μM) in a medium in which Ca^2+^ was replaced by Sr^2+^; or (iii) RPMI alone (control) (**C**) Time-course of CB-induced LD formation. Macrophages were incubated with CB (0.4 µM) or RPMI (control) for 1, 3, 6, 12 or 24 h. LDs were quantified using light microscopy after OsO_4_ staining. (**D**) Osmium-stained LDs observed in control cells or in cells stimulated with CB (0.4 µM) for 1 h (**E**), 6 h (**F**) or 12 h (**G**). Each bar represents the mean ± SEM of the number of LDs/cell in 50 cells. Values represent mean ± SEM for three to five animals. *p < 0.05 compared with RPMI-stimulated cells; ^&^p < 0.05 compared with cells stimulated with CB for 1 h, ^#^p < 0.05 compared with cells stimulated with CB for 1, 3 and 6 h, °p < 0.05 compared with cells stimulated with CB for 12 h, and ^+^p < 0.05 compared with CB/Ca^2+^-stimulated cells.

To investigate whether CB would induce formation of LDs in isolated macrophages, these cells were incubated for 1 h with CB at non cytotoxic concentrations ranging from 0.1 to 0.8 µM. Lack of cytotoxicity with these concentrations of CB was previously determined (1–24 h time intervals) by MTT assay. As shown in Fig. 1A, incubation of macrophages for 1 h with CB at concentrations ranging from 0.2 to 0.8 µM, but not 0.1 µM, induced a significant increase in the number of LDs compared with control cells incubated with culture medium alone. Maximal LD numbers were observed with a concentration of 0.8 µM. This CB-induced effect was not observed in Ca^2+^-free, EGTA-containing medium, in a medium in which Ca^2+^ had been replaced by Sr^2+^ or when CB was inactivated by p-bromophenacyl bromide (p-BPB) (Fig. 1B), indicating that the catalytic activity of this sPLA_2_ is essential for induction of LD formation under the present experimental conditions. To determine the time-course of CB-induced LD formation, a submaximal concentration of this sPLA_2_ was used (0.4 µM), and the number of LDs after 1–24 h of incubation was determined. As shown in Fig. 1, CB caused a significant increase in the number of LDs after 1–24 h incubation compared with control cells. The greatest number of LDs was detected after 12 h of incubation (Fig. 1C). As illustrated in Fig. 1D, control macrophages stained with OsO_4_ showed very few inclusions in the cytoplasm. In contrast, the cytoplasm in CB-stimulated macrophages was packed with the osmiophilic organelles, which can be seen as dark punctate structures in Fig. 1E, F and G. Taken together, these findings indicate that CB is able to induce LD biogenesis in cultured macrophages in a rapid-onset effect that is dependent on the incubation time and catalytic activity of this enzyme.

### CB induces protein expression of PLIN2 and its recruitment to LDs in macrophages

**Figure 2:**
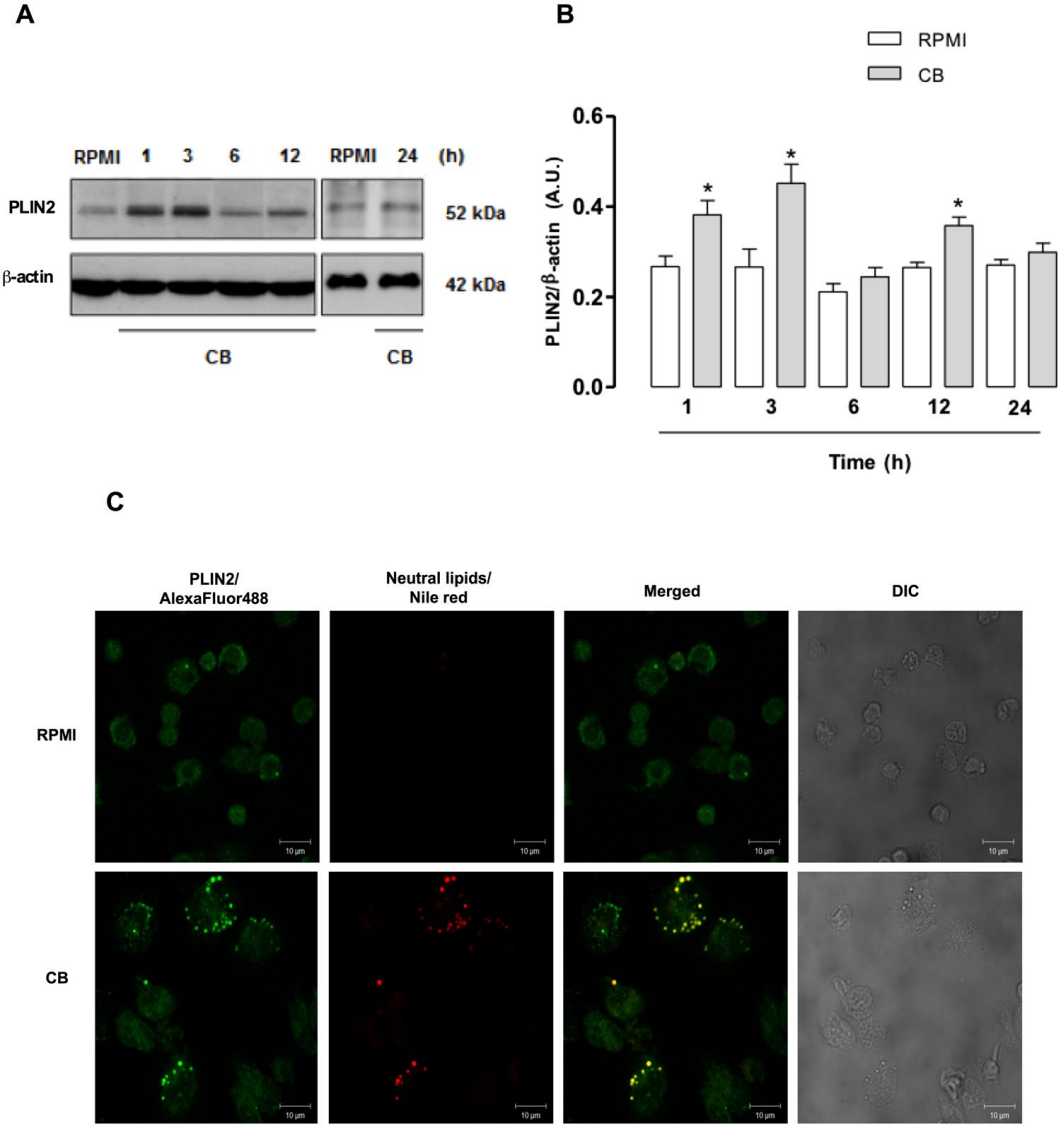
CB up- regulates PLIN2 protein expression in macrophages. Peritoneal macrophages were incubated with CB (0.4 µM) or RPMI (control) for 1, 3, 6, 12 and 24 h. (**A**) Western blotting of PLIN2 and β-actin (loading control) in macrophage extracts. Immunoreactive bands of 24 h were obtained in a distinct assay. (**B**) Densitometric analysis of PLIN2 bands. The densities (in arbitrary units) were normalized with those of β-actin. Results are expressed as mean ± SEM for three experiments. *p < 0.05 compared with RPMI. Blots were cropped and full-length blots can be found as Supplementary Figure S1. (**C**) Macrophages were incubated with RPMI (control) or CB (0.4 µM) for 3 h and labeled for LDs (with Nile Red) and for PLIN2 (with FITC-conjugated antibody). The merged image shows colocalization of PLIN2 to LDs. The pictures are representative of three independent experiments.

PLIN2 is a fatty acid-binding protein that plays an important role in LD assembly and the development of foamy macrophages^18^. In an attempt to better understand the stimulatory effect of CB that leads to LD formation in macrophages, we investigated whether CB induces expression of PLIN2. While this revealed increased levels of PLIN2 expression in cell extracts stimulated with CB for 1, 3 and 12 h, but not for 6 and 24 h, extracts from unstimulated cells (controls) failed to show significant levels of PLIN2 for any of the incubation periods tested (Fig. 2A, B and Supplementary Fig. S1). These findings demonstrate that CB is able to up-regulate PLIN2 expression at translational levels and suggest a mechanism for the increase in LB formation detected in CB-stimulated macrophages. Considering that the increase of PLIN2 protein expression occurred since the first hour of stimulation by CB it is possible that the amount of PLIN2 needed to supply the increased assembly of LDs induced by CB was enough at 6 h of stimulation leading to the transient lack of increase of PLIN2 protein expression seen at 6 h of stimulation by this sPLA_2_. At 24 h, however, the absence of PLIN2 protein expression was accompanied by a significant decay of LD numbers, suggesting that CB-induced effect falls into decline. To further identify the mechanisms involved in CB-induced LD formation, the ability of CB to recruit PLIN2 into cytosol LDs was investigated. As illustrated in Fig. 2C, macrophages stimulated with CB (0.4 µM) for 3 h exhibited strong fluorescent staining (green) for PLIN2 with a punctate cytoplasmic pattern that was absent in the unstimulated control cells. Fluorescent Nile Red-labeled neutral lipid inclusions (LDs) overlapping with stained cytoplasmic PLIN2 were also visualized 3 h after CB-induced stimulation indicating that PLIN2 co-localizes to LDs. As expected, no significant staining was detected in control macrophages. These data demonstrate the ability of CB to recruit PLIN2 from cell membranes to form new LDs in macrophages.

### Distinct signaling pathways are involved in CB-induced LD formation

**Figure 3:**
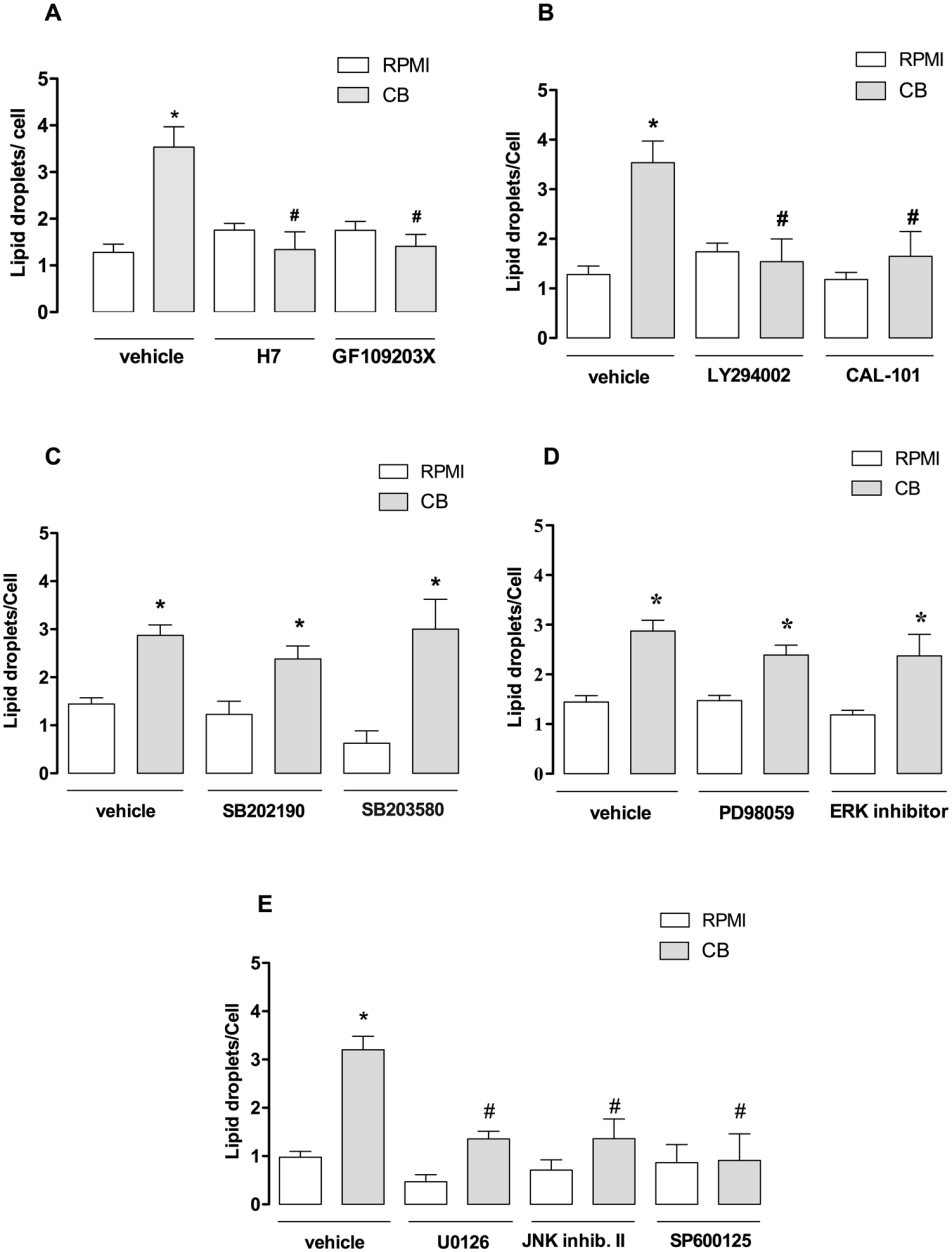
Signaling pathways involved in CB-induced LD formation in macrophages. Macrophages were incubated with one of the following inhibitors before stimulation with CB (0.4 µM) for 3 h: (**A**) PKC inhibitors H7 (6 µM) or GF109203X (1 μM) for 1 h, (**B**) PI3K inhibitors LY294002 (1 µM) or CAL101 (1 µM) for 1 h; (**C**) p38^MAPK^ SB inhibitors SB201290 (5 µM) or SB203580 (16 µM); (**D**) ERK1/2 inhibitors PD98059 (25 µM) or ERK inhibitor (5 µM) for 1 h; (**E**) MEK1/2 inhibitor, U0126 (25 µM) or JNK inhibitors JNK inhibitor II (2 μM) or SP600125 (10 µM) for 1 h. LDs were counted using light microscopy after osmium staining. Each bar represents the mean ± SEM of the number of LDs/cell in 50 cells. Values represent means ± SEM for three to five animals. *p < 0.05 compared with RPMI/vehicle-stimulated cells; ^#^ *p* < 0.05 compared with vehicle treated/CB-stimulated cells.

**Figure 4:**
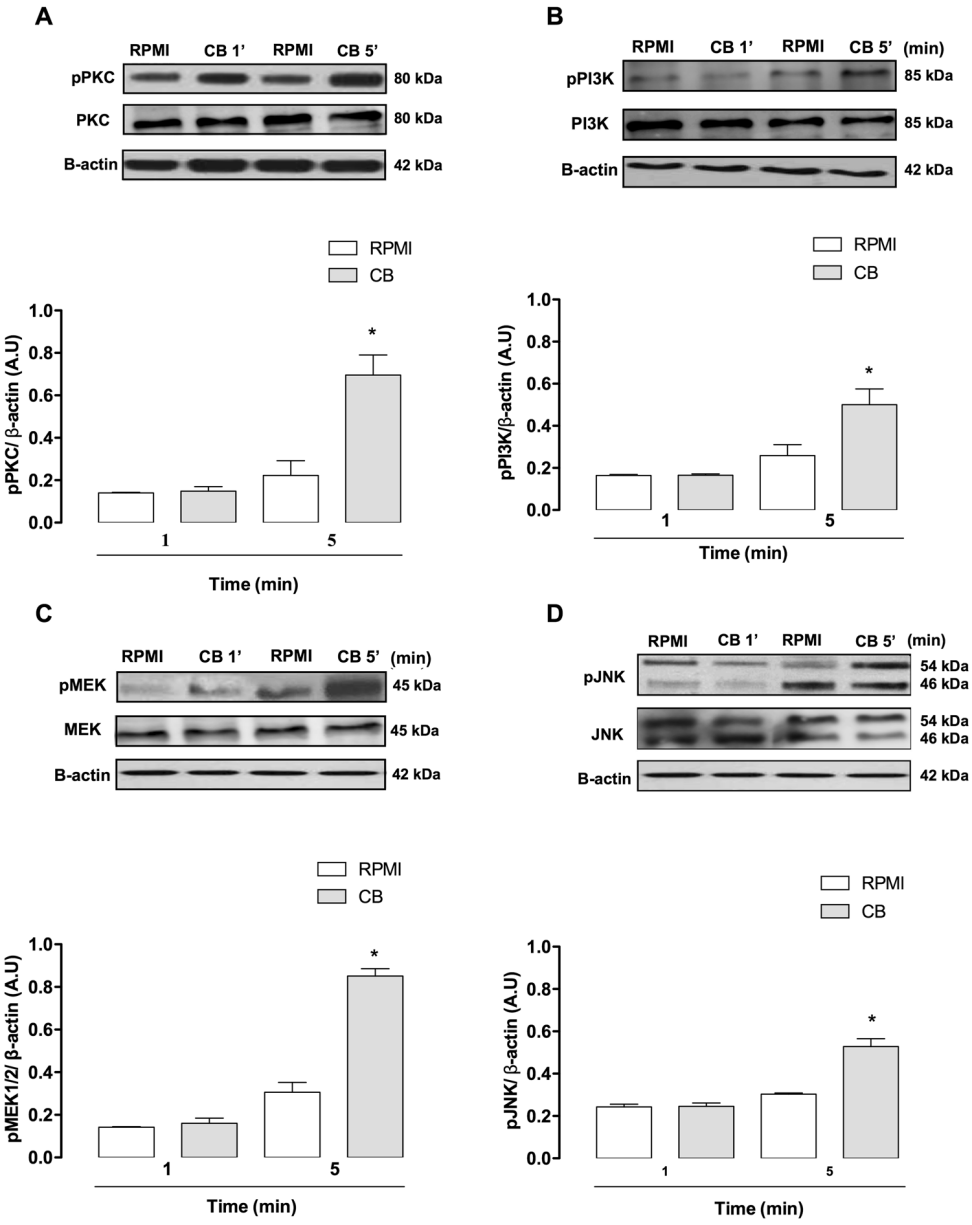
CB induces PKC, PI3K, MEK1/2 and JNK activation in isolated macrophages. Macrophages were incubated with CB (0.4 μM) or RPMI (control) for 1 or 5 min. Western blotting of total and phospho-PKC (**A**), total and phospho- PI3K (**B**), total and phospho-MEK1/2 (**C**), total and phospho-JNK (**D**) and β-actin (loading control) in macrophage extracts. Densitometric analysis of the phosphorylated protein bands (**E**, **F**, **G** and **H**). The densities (in arbitrary units) were normalized with those of β-actin. Results are expressed as mean ± SEM for three experiments. *p < 0.05 compared with RPMI-stimulated cells.

Regulation of LD formation induced by diverse inflammatory agents involves stimulus-specific signaling pathways^46^. Participation of protein kinases in the signaling triggered by sPLA_2_s has been previously reported^47, 48^. Therefore, the critical signaling proteins involved in CB-induced LD biogenesis were investigated using pharmacological approaches. The effects of selected pharmacological treatments were evaluated 3 h after incubation of macrophages with CB. To investigate the role of PKC and PI3K in CB-induced LD formation, macrophages were treated with selective PKC and PI3K inhibitors (H7, GF109203X and LY294002, CAL101, respectively) or their vehicles (controls) prior to incubation with CB, followed by quantification of osmium-stained LDs. As seen in Fig. 3A and B, both PKC inhibitors (H7 and GF109203X) and PI3K inhibitors (LY294002 and CAL101) abolished the increased LD formation in CB-stimulated macrophages compared with vehicle-treated CB-stimulated macrophages. Next, we investigated whether the effect of CB on LD formation is related to MAPKs p38^MAPK^, ERK1/2 or JNK. As demonstrated in Fig. 3C and D, neither ERK1/2 inhibitors **(**PD98059 and ERK inhibitor), nor p38^MAPK^ inhibitors (SB202190 and SB203580) changed the number of LDs in CB-stimulated macrophages compared with vehicle-treated macrophages stimulated with CB. Furthermore, Fig. 3E shows that pharmacological inhibition of cells with JNK inhibitors (JNK inhibitor II and SP600125) abrogated LD formation in CB-stimulated macrophages compared with untreated control cells. The next protein evaluated was MEK1/2, an important upstream protein of the MAPK signaling pathway. U0126, a MEK1/2 inhibitor, reduced the increase in LD formation in CB-stimulated macrophages compared with vehicle-treated control cells (Fig. 3E), reinforcing the role of the MAPK family in CB-induced LD formation in macrophages. Activation of the proteins PKC, PI3K, MEK1/2 and JNK was confirmed by evaluating the extent of phosphorylation (Fig. 4A, B, C and D). CB induced a significant increase in phosphorylation of these proteins only at the 5-minute time point.

### PLD and iPLA_2_ are involved in CB-induced LD formation in macrophages

**Figure 5:**
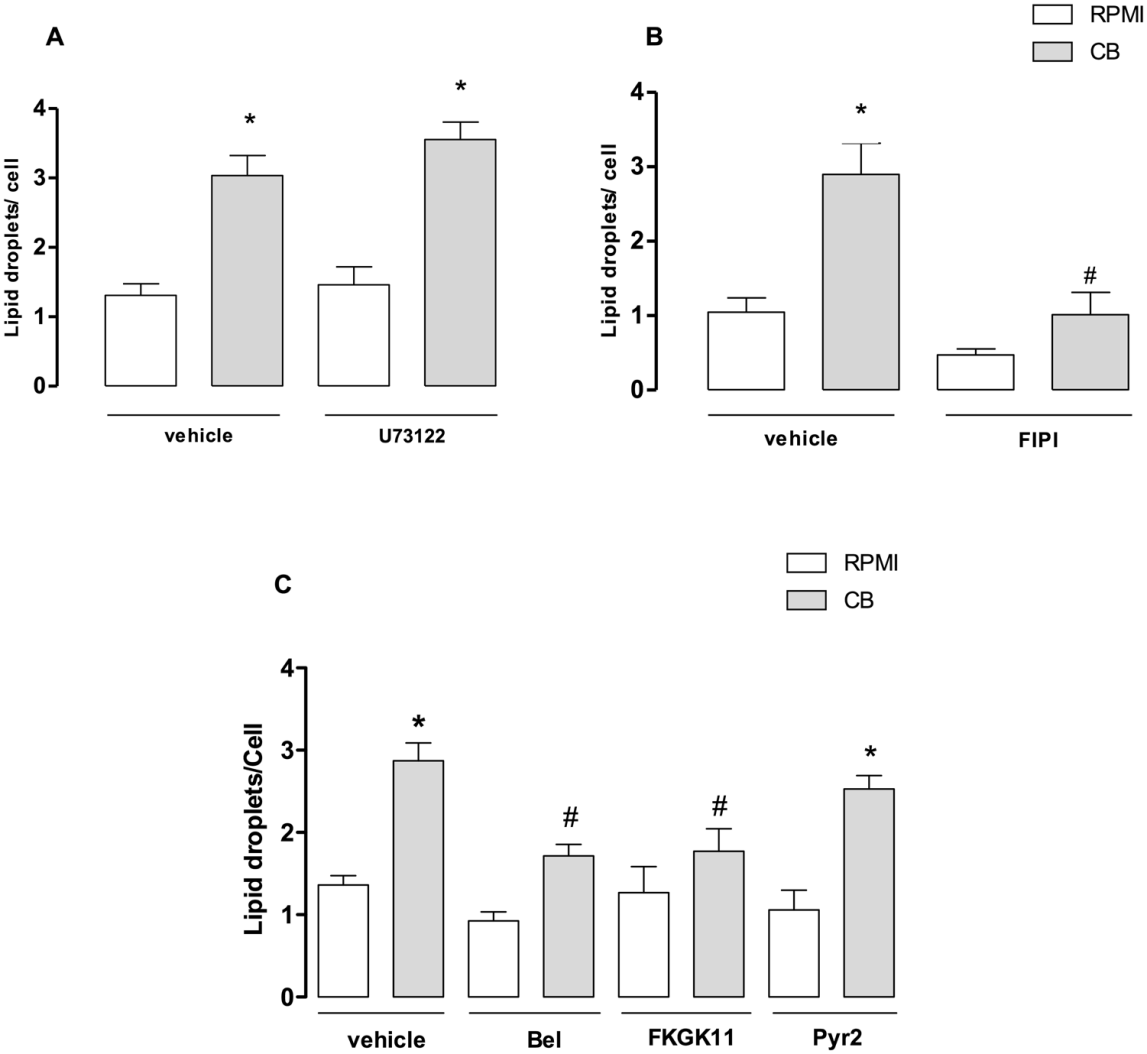
PLD and iPLA_2_, but not PLC and cPLA_2_ are required for CB-induced LD formation. Peritoneal macrophages were incubated with the inhibitors U73122-1 µM (**A**), FIPI-1 µM (**B**), BEL-2 µM, FKGK11- 1 µM or Pyr-2-1 µM (**C**) for 30 min and then with CB (0.4 µM) for 3 h. LDs were quantified using light microscopy after osmium staining. Each bar represents the mean ± SEM of LDs/cell in 50 cells. Values represent means ± SEM for 3–5 animals. *p < 0.05 compared with RPMI/vehicle-stimulated cells; ^#^ *p* < 0.05 compared with vehicle treated/CB-stimulated cells.

It has been reported that phospholipase D (PLD) and phospholipase C (PLC) play important roles in the signal transduction pathways associated with LD formation^49^. As seen in Fig. 5A, pre-treatment of macrophages with U73122, an inhibitor of PLC, did not have an effect on CB-induced LD formation in comparison with control cells. However, when the cells were incubated with compound FIPI, an inhibitor of PLD (Fig. 5B), LD formation induced by CB was abrogated compared with controls. These results indicate that PLD is critical for the stimulatory effect of CB on LDs formation in macrophages, whereas PLC does not contribute to this CB-induced effect. Additionally, it is known that the intracellular PLA_2_s, such as calcium independent PLA_2_ (iPLA_2_) and cytosolic PLA_2_ (cPLA_2_) have a role in LDs formation^47, 50^. Moreover, a cross talk between sPLA_2_s and intracellular PLA_2_s for production of prostaglandins and LDs has been described^7, 47^. Therefore, to verify whether iPLA_2_ and/or cPLA_2_ would be committed to CB-induced LD formation, macrophages were treated with low micromolar concentrations of Pyr-2, a specific inhibitor of cPLA_2_α, or with either BEL or FKGK11, both inhibitors of iPLA_2_. As shown in Fig. 5C, treatment of macrophages with BEL or FKGK11, but not Pyr-2, caused a significant reduction in the number of LDs in CB-stimulated macrophages compared with CB-stimulated vehicle-treated cells. These data indicate that iPLA_2_, but not cPLA_2_ plays a role in LD formation induced by CB in macrophages.

### CB induces production of 15-d-PGJ_2_ via COX-1 in macrophages

**Figure 6:**
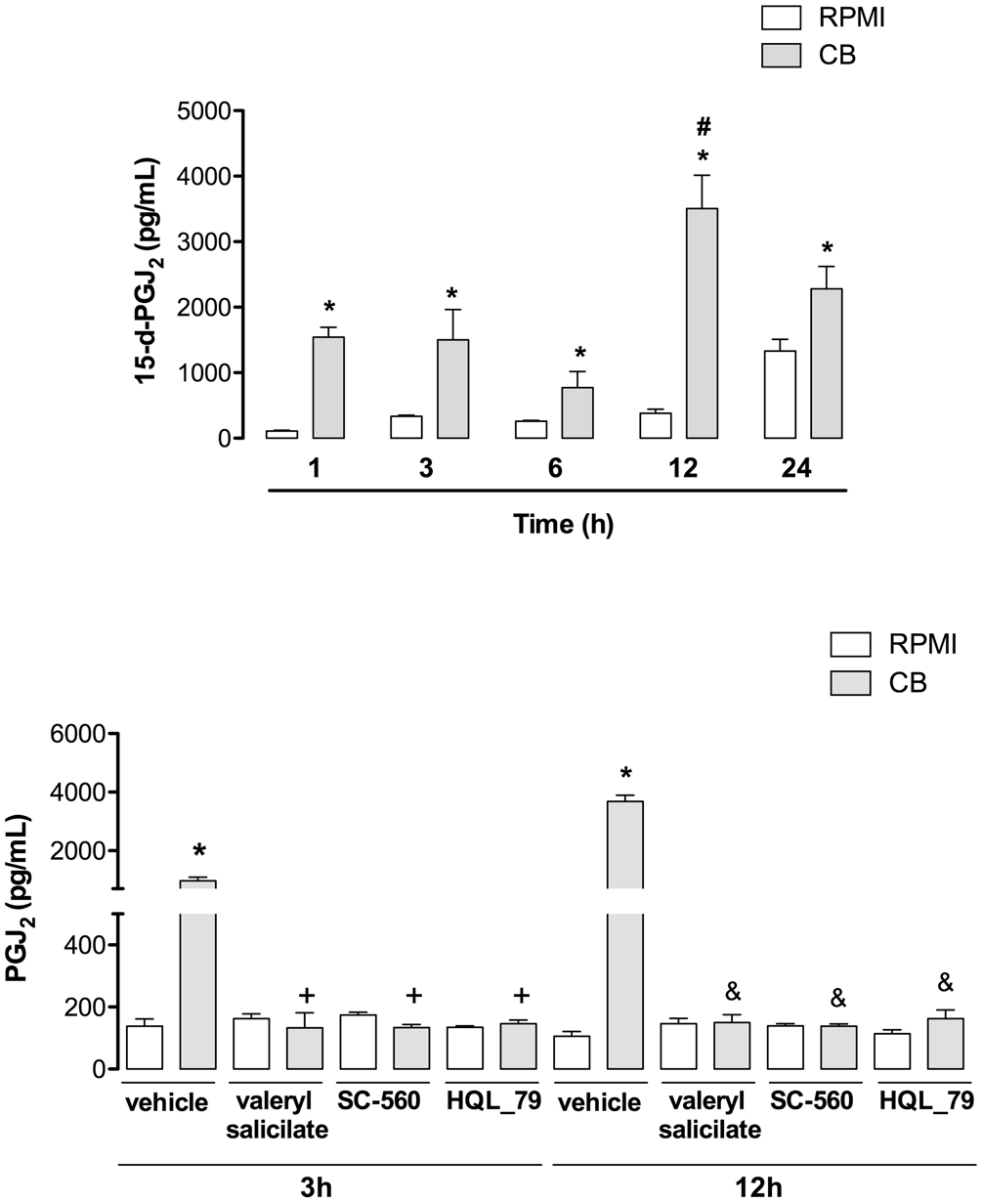
CB induces COX-1- and PGDS-dependent release of 15-d-PGJ_2_ from macrophages. Macrophages (1 × 10^6^ cells) were incubated with CB (0.4 µM) or RPMI (control) for 1 up to 24 h. (**A**) Quantification of 15-d-PGJ_2_ in culture supernatants by specific EIA. (**B**) Treatment of macrophages with valeryl salicylate (450 μM), SC-560 (0.5 μM) or HQL-79 (30 μM) for 1 h before stimulation with CB (0.4 µM) or RPMI (control) for 3 or 12 h. 15-d-PGJ_2_ was quantified by specific EIA. Values represent means ± SEM from 3–4 experiments. *p < 0.05 as compared with RPMI/vehicle-stimulated cells, ^+^p < 0.05 and ^ &^p < 0.05 as compared with vehicle treated/CB-stimulated cells, and ^#^p < 0.05 as compared with CB-stimulated cells for 1, 3, 6 and 24 h.

15-d-PGJ_2_, which is a PGD_2_-derived prostanoid^51^, has been described as a pro-resolving lipid mediator, which is involved in the switch from the acute into the regenerative phases of inflammation^52^. We have previously reported that CB elicits production of high levels of PGD_2_ by isolated macrophages^7^. On this basis, we evaluated the ability of CB to induce the release of 15-d-PGJ_2_ from macrophages in culture. 15-d-PGJ_2_ concentration was measured in supernatants from macrophages incubated with either a sub-cytotoxic concentration of CB (0.4 μM) or RPMI alone (control). As shown in Fig. 6A, incubation of macrophages with CB induced a significant increase in 15-d-PGJ_2_ concentration in the supernatants from 1 up to 24 h, with levels ranging from 1545 ± 149.5 to 2283 ± 340.5 pg/mL (*p* < 0.05). The corresponding control values ranged from 110.1 ± 11.5 to 1333 ± 179.3 pg/mL. These results are evidence of the ability of CB to induce generation of 15-d-PGJ_2_. Considering that (i) cyclooxygenases are key enzymes in the prostaglandin biosynthetic pathway and (ii) previous data from our group showing that COX-1, but not COX-2 is activated by CB in macrophages^7^, the effect of inhibitors of COX-1 on CB-induced biosynthesis of 15-d-PGJ_2_ was examined. As demonstrated in Fig. 6B, 1 h pretreatment of macrophages cultures with valeryl salicylate (450 μM) or SC-560 (0.5 μM), both specific COX-1 inhibitors, abolished 15-d-PGJ_2_ release induced by CB after 3 h and 12 h of incubation in comparison with control cells, pretreated with vehicle and stimulated with CB, indicating participation of COX-1 isoform in the synthesis of 15-d-PGJ_2_ induced by CB. In addition, we evaluated the participation of PGD synthase coupled to COX-1 in CB-induced 15-d-PGJ_2_ release. As shown in same Fig. (6B), 1 h pretreatment of macrophages cultures with HQ compound (30 μM), a prostaglandin D synthase (PGDS) inhibitor, abolished 15-d-PGJ_2_ release induced by CB at 3 and 12 h of incubation in comparison with control cells, pretreated with vehicle and stimulated with CB, confirming the role of PGD_2_ as the precursor of 15-d-PGJ_2_ in the present experimental condition.

### Cytoplasmic LDs in CB-stimulated macrophages compartimentalize 15-d-PGJ_2_ and COX-1 and drive 15-d-PGJ_2_ production

**Figure 7:**
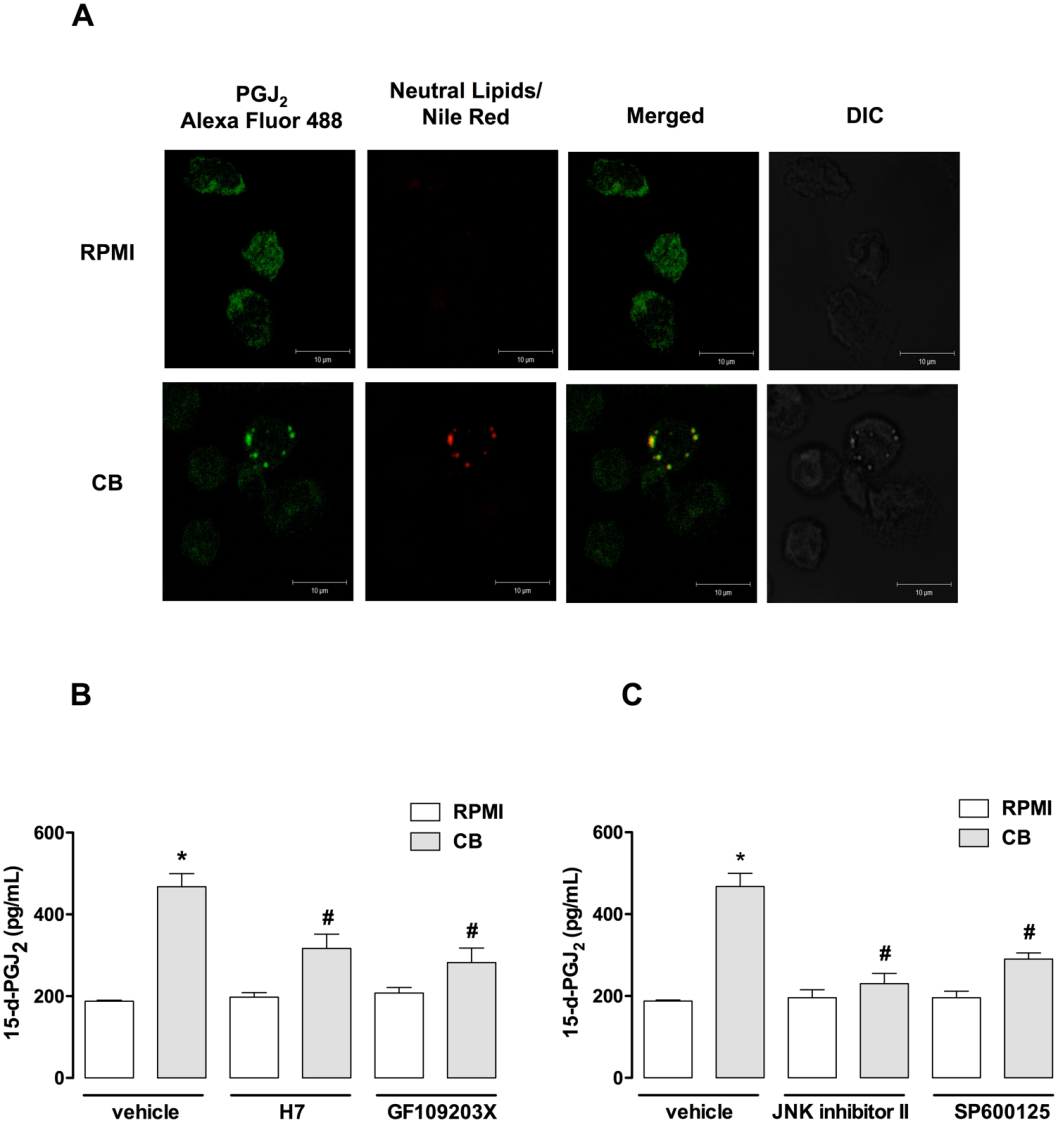
CB-induced cytoplasmic LDs compartmentalize 15-d-PGJ_2_. (**A**) Macrophages incubated with RPMI (control) or CB (0.4 µM) for 3 h were labeled for LDs (Nile Red) and for 15d-PGJ_2_ (anti-15-d-PGJ_2_ antibody, Cayman Chemical). The merged image shows colocalization of 15-d-PGJ_2_ to LDs. The pictures are representative of three independent experiments. (**B**) Treatment of macrophages with PKC inhibitors H7 (6 µM) or GF109203X (1 μM) or (C) with JNK inhibitors JNK inhibitor II (2 μM) or SP600125 (10 µM) for 1 h before stimulation with CB (0.4 µM) or RPMI (control) for 3 h. 15-d-PGJ_2_ was quantified by specific EIA. Values represent means ± SEM from 3–4 experiments. *p < 0.05 as compared with vehicle treated RPMI-stimulated cells, ^#^ *p* < 0.05 as compared with vehicle treated CB-stimulated cells.

**Figure 8:**
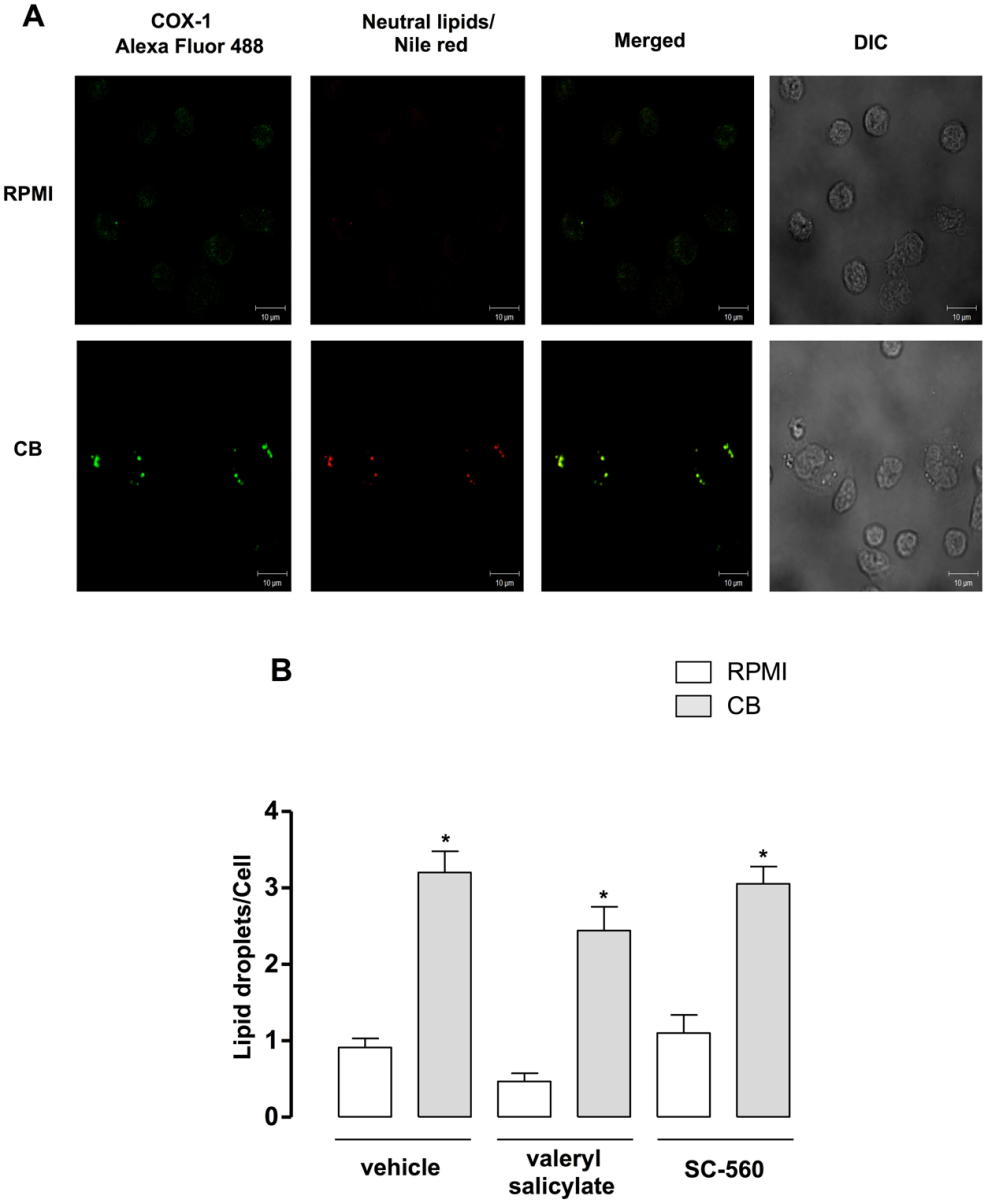
CB-induced cytoplasmic LDs compartmentalize COX-1, but this enzyme is not involved in LDs formation. (**A**) Macrophages incubated with RPMI (control) or CB (0.4 µM) for 3 h were labeled for LDs (Nile Red) and for COX-1 (anti-COX-1 antibody, Cayman Chemical). The merged image shows colocalization of COX-1 to LDs. The pictures are representative of three independent experiments. (**B**) Treatment of macrophages with valeryl salicylate (450 μM) or SC-560 (0.5 μM) a COX-1 inhibitors for 1 h, before stimulation with CB (0.4 µM) for 3 h. LDs were counted using light microscopy after osmium staining. Each bar represents the mean ± SEM of the number of LDs/cell in 50 cells. Values represent means ± SEM for three to five animals. *p < 0.05 compared

LDs are known to be organelles associated with the synthesis and storage of inflammatory mediators in immunocompetent cells, such as macrophages. In order to investigate the possible role of CB-induced LDs as intracellular sites for the synthesis of lipid mediators, specially 15-d-PGJ_2_, macrophages stimulated or not by CB were incubated with EDAC to immobilize the newly synthesized eicosanoids^45^ and immunostained with antibodies against 15-d-PGJ_2_ and against COX-1, and LDs were stained with Nile Red. As illustrated in Fig. 7A, macrophages stimulated with CB (0.4 µM) for 3 h exhibited a (green) cytoplasmic staining pattern for 15-d-PGJ_2_. This pattern was not observed in the unstimulated control cells, which exhibited diffuse staining. Fluorescent Nile Red-labeled LDs were also visualized 3 h after stimulation by CB and were virtually absent in unstimulated control macrophages. Overlapping images show that stained cytoplasmic 15-d-PGJ_2_ matched perfectly with neutral lipid inclusions in CB-stimulated macrophages indicating that 15-d-PGJ_2_ co-localizes to LDs. In light of our present results showing that formation of LDs induced by CB was inhibited by PKC and JNK, the role of LDs in the synthesis of 15-d-PGJ_2_ induced by CB was further investigated pretreating macrophages with either PKC inhibitors (H7 and GF109203X) or JNK inhibitors (JNK inhibitor II and SP600125), which abolished CB-induced LD formation, or their vehicles (controls) prior to incubation with CB for 3 h, followed by quantification of 15-d-PGJ_2_ levels in cell supernatants. As seen in Fig. 7B, treatment of cells with PKC inhibitors abolished the increased 15-d-PGJ_2_ production in CB-stimulated macrophages compared with vehicle-treated CB-stimulated macrophages. Moreover, Fig. 7C demonstrates that pharmacological inhibition of cells with JNK inhibitors abrogated 15-d-PGJ_2_ production in CB-stimulated macrophages compared with untreated control cells. In addition, as illustrated in Fig. 8A, macrophages stimulated with CB (0.4 µM) for 3 h exhibited a punctate cytoplasmic fluorescence (green) for COX-1. This pattern could not be observed in the unstimulated control cells, in which diffuse staining was observed. Fluorescent Nile Red-labeled LDs were also visualized 3 h after stimulation by CB and were virtually absent in unstimulated control macrophages. Overlapping images show that stained cytoplasmic 15-d-PGJ_2_ and COX-1 matched perfectly with neutral lipid inclusions in CB-stimulated macrophages. Considering these results we investigated whether COX-1 contributes to formation of LDs induced by CB. As demonstrated in Fig. 8B, 1 h pretreatment of macrophages cultures with valeryl salicylate (450 μM) or SC-560 (0.5 μM), both specific COX-1 inhibitors, did not affect LDs formation induced by CB after 3 h of stimulation in comparison with control cells, pretreated with vehicle and stimulated with CB, indicating that COX-1 isoform is not involved in formation of LDs induced by CB. In the present experimental condition, this isoform is committed to 15-d-PGJ_2_ synthesis. Taken together these findings indicate that CB has the capability to induce the synthesis of a pro-resolving lipid mediator (15-d-PGJ_2_) and its compartimentalization, in association with the prostaglandin-forming enzyme COX-1, in LDs. These data indicate the role of LDs as a relevant site for the synthesis and accumulation of 15-d-PGJ_2_ in macrophages stimulated by CB.

## Discussion

Group IIA secreted phospholipases A_2_ are enzymes whose proinflammatory properties have been well established^53, 54^. However, CB, a group IIA sPLA_2_ isolated from *Cdt* snake venom, interferes with the development of the inflammatory response by blocking important stages in this process, such as leukocyte recruitment and macrophage activation^4, 5^. In the present study, the ability of this GIIA sPLA_2_ to directly stimulate formation of LDs in macrophages and the mechanisms involved in this phenomenon were demonstrated. This effect was time-dependent, had a very fast onset with a maximum at 12 h, and persisted up to the 24-hour time-point. These data agree with previous reports from our group showing that two proinflammatory secreted PLA_2_s from *Bothrops* snake venom are able to induce LD formation in isolated macrophages^47, 48^. These results are interesting as formation of this organelle in leukocytes is generally associated with a variety of phlogistic stimuli^13, 55, 56^, including stimuli by proinflammatory group IIA sPLA_2_s^47, 48^. In addition, to our knowledge, this is the first study showing that LD formation can be induced by group IIA sPLA_2_ that does not induce an inflammatory response.

PLIN2 is a LD marker protein and plays an important role in LD assembly and foam cell formation^18–20^ by its properties to bind lipids with high affinity and increase the uptake of long chain fatty acids^57^. Several studies have shown that PLIN2 expression has been directly related to enhanced neutral lipid storage and biogenesis of LDs in a variety of cell types^13, 21, 46, 47^. Our results show that CB induces PLIN2 recruitment from its membrane pools, suggesting a role for this protein as a nucleation site for formation of new LDs under the stimulus by this sPLA_2_. Moreover, up-regulation of PLIN2 expression observed within 12 h of stimulation by CB suggests a mechanism for LD formation in CB-stimulated macrophages. These results differ from those obtained with MT-III, a pro-inflammatory PLA_2_ isolated from *Bothrops* snake venom, which induced an increase in PLIN2 protein expression in macrophages only 6 h after they were stimulated^47^.

Next, we investigated the mechanisms by which CB stimulates LD formation in macrophages by focusing on major downstream signaling protein kinases that have previously been shown to participate in LD biogenesis induced by inflammatory and infectious stimuli. Our results revealed rapid activation of the signaling proteins PKC, PI3K, MEK1/2 and JNK, but not p38^MAPK^ or ERK1/2, in macrophages activated by CB. While inhibition of PKC, PI3K, MEK1/2 and JNK abolished the CB-induced increase in LD formation, indicating that these signaling proteins contribute to this increase, p38^MAPK^ and ERK1/2 do not play a role in CB-induced LD formation. In light of these results, it is reasonable to suggest that PKC and PI3K are crucial in LD formation induced by venom sPLA_2_s since both proteins are involved in LD formation induced by both *Crotalus* (CB) and *Bothrops* sPLA_2_s (MT-III and MT-II)^47, 48^. Our finding that CB-induced LD formation requires activation of the PI3K pathway is in accordance with data in the literature showing that this signaling protein plays a key role in pathways that up-regulate proteins associated with lipid accumulation such as peroxisome proliferator-activated receptor-gamma (PPAR-γ) and PLIN2^58, 59^. In this context, our finding that CB-induced LD formation in macrophages is largely dependent on both the MEK1/2 and JNK pathways agrees with the literature, which indicates that MEK1/2 and JNK are important signaling proteins implicated in foam-cell formation^60, 61^.

The literature shows that group IIA sPLA_2_s interact with intracellular PLA_2_s in the biosynthesis of lipid mediators from fatty acids^6^ and also that LD formation involves the activation of intracellular PLA_2_s, such as iPLA_2_ and cPLA_2_, as well as other classes of intracellular phospholipases, including PLC and PLD^47–50^. Our finding that inhibition of iPLA_2_ by BEL and FKGK11 reduced CB-induced LD biogenesis indicates that this intracellular PLA_2_ contributes to the effect of CB. However, although cPLA_2_ has been reported to be relevant to LDs formation induced by diverse PAMPS stimuli and a snake venom inflammatory sPLA_2_^25, 47^ this intracellular PLA_2_ does not participate in the genesis of LDs induced by CB in macrophages. A recent study showed the important role of iPLA_2_ in fatty acid metabolism and triacylglycerol formation^62^. Since CB induces the release of high levels of arachidonic acid in macrophages^7^, our data suggest that upon stimulation by CB this fatty acid contributes to the synthesis of triacylglycerol, which in turn is an important component of LDs. However, the mechanism by which CB activates iPLA_2_ is unknown and will be investigated in future studies by our group. Additionally, our data show that PLD, but not PLC, is an important step in the signaling pathways involved in the CB-induced effect. It has been shown that PLD acts on the membrane lipid phosphatidylcholine to produce the signal molecule phosphatidic acid (PA), which is a negatively charged phospholipid whose small headgroup promotes membrane curvature, allowing lipid accumulation at this local and facilitating the budding of LDs^63^. In addition, PA can be converted to bioactive lipids, such as diacylglycerol and lysophosphatidic acid^64^. Therefore, any or all of these mechanisms may act in conjunction to mediate LD formation. This is the first demonstration that a group IIA sPLA_2_ crosstalks with PLD, activating LD formation. This finding agrees with an earlier study which showed that activation of PLD_2_ was induced by a murine group IIA sPLA_2_, indicating that there is crosstalk between PLA_2_s and PLD^65^.

A previous study by our group showed that CB induces production of high levels of PGD_2_ by macrophages^7^. It is known that PGD_2_ can be chemically modified by non-enzymatic dehydration reactions, leading to production of the cyclopentenone prostaglandin 15-deoxy-Δ^12, 14^ PGJ_2_ (15-d-PGJ_2_), an important lipid mediator involved in the resolution phase of inflammation^11, 12^. In line with our previous findings, the results of the present study show that CB-stimulated macrophages release significant amounts of 15-d-PGJ_2_ at all the time points, in a process dependent on COX-1 pathway and the terminal PGDS. Furthermore, our data show that **(**i) CB-induced LDs compartmentalizes both COX-1 and 15-d-PGJ_2_ and (ii) CB-induced 15-d-PGJ_2_ is abrogated when LD formation is inhibited by pretreatment of macrophages with inhibitors of PKC and JNK, upstream and downstream signaling proteins, respectively, that were shown to inhibit LD formation induced by CB. In light of these data, and considering that COX-1, but not COX-2, is activated by CB in macrophages^7^, and as LDs are important organelles for eicosanoid synthesis^66, 67^, it is reasonable to suggest that CB-induced LDs are sites for 15-d-PGJ_2_ synthesis via the COX-1 enzymatic system. To our knowledge, this is the first demonstration that LDs induced by a group IIA sPLA_2_ are equipped with a COX-1 enzymatic system and are involved in synthesis of a lipid mediator implicated in inflammatory resolution, such as 15-d-PGJ_2_. In addition, it should be noted that release of the anti-inflammatory mediator 15-d-PGJ_2_ is in line with the immunomodulatory effects of CB and sheds new light on the mechanisms by which whole *Crotalus* snake venom and CB, its major sPLA_2_, can reduce or abolish inflammatory events^5, 68^.

In conclusion, our data show that group IIA sPLA_2_ CB directly activates peritoneal macrophages to form LDs by a mechanism that is dependent on increased expression and distribution of PLIN2 and requires activation of PKC, PI3K, MEK1/2 and JNK. We also showed that this venom sPLA_2_ crosstalks with iPLA_2_ and PLD to induce LDs formation. Our results also indicate for the first time that LDs can constitute platforms implicated in the synthesis of anti-inflammatory lipid mediators such as 15-d-PGJ_2_ in macrophages stimulated by a sPLA_2_. Therefore, the LDs, classicaly recognized as relevant organelles of the acute phase of inflammation, can be involved in both development and resolution of the inflammatory process. Finally, our results reinforce previous findings that phospholipases A_2_ from snake venom can induce LDs formation in immunocompetent cells and afford new insights into the roles of LDs in the inflammatory process.

## Electronic supplementary material

Supplementary Figure 1
