# Supplementary material for: A snake venom group IIA PLA2 with immunomodulatory activity induces formation of lipid droplets containing 15-d-PGJ2 in macrophages
Source: Sci Rep. 2017 Jun 22;7:4098. doi: 10.1038/s41598-017-04498-8 (PMC5481388; doi:10.1038/s41598-017-04498-8)
Supplement: Supplementary file 1 — Supplementary Figure 1 [file 41598_2017_4498_MOESM1_ESM.doc]

**Suplemmentary data**

A snake venom group IIA PLA_2_ with immunomodulatory activity induces formation of lipid droplets containing 15-d-PGJ_2_ in macrophages

Karina Cristina Giannotti^1†^, Elbio Leiguez^1†^, Ana Eduarda Zulim de Carvalho^1^, Neide Galvão Nascimento^1^, Márcio Hideki Matsubara^1^, Consuelo Latorre Fortes-Dias^2^, Vanessa Moreira^3^, Catarina Teixeira^1*^

^1^Pharmacology Laboratory, Butantan Institute, São Paulo, SP, Brazil.

^2^Molecular Biology and Bioinformatics Laboratory, Ezequiel Dias Foundation, Belo Horizonte, MG, Brazil.

^3^Department of Pharmacology, Paulista School of Medicine, Federal University of São Paulo, São Paulo, CEP 04044-020, Brazil.

† These authors contributed equally to this work.


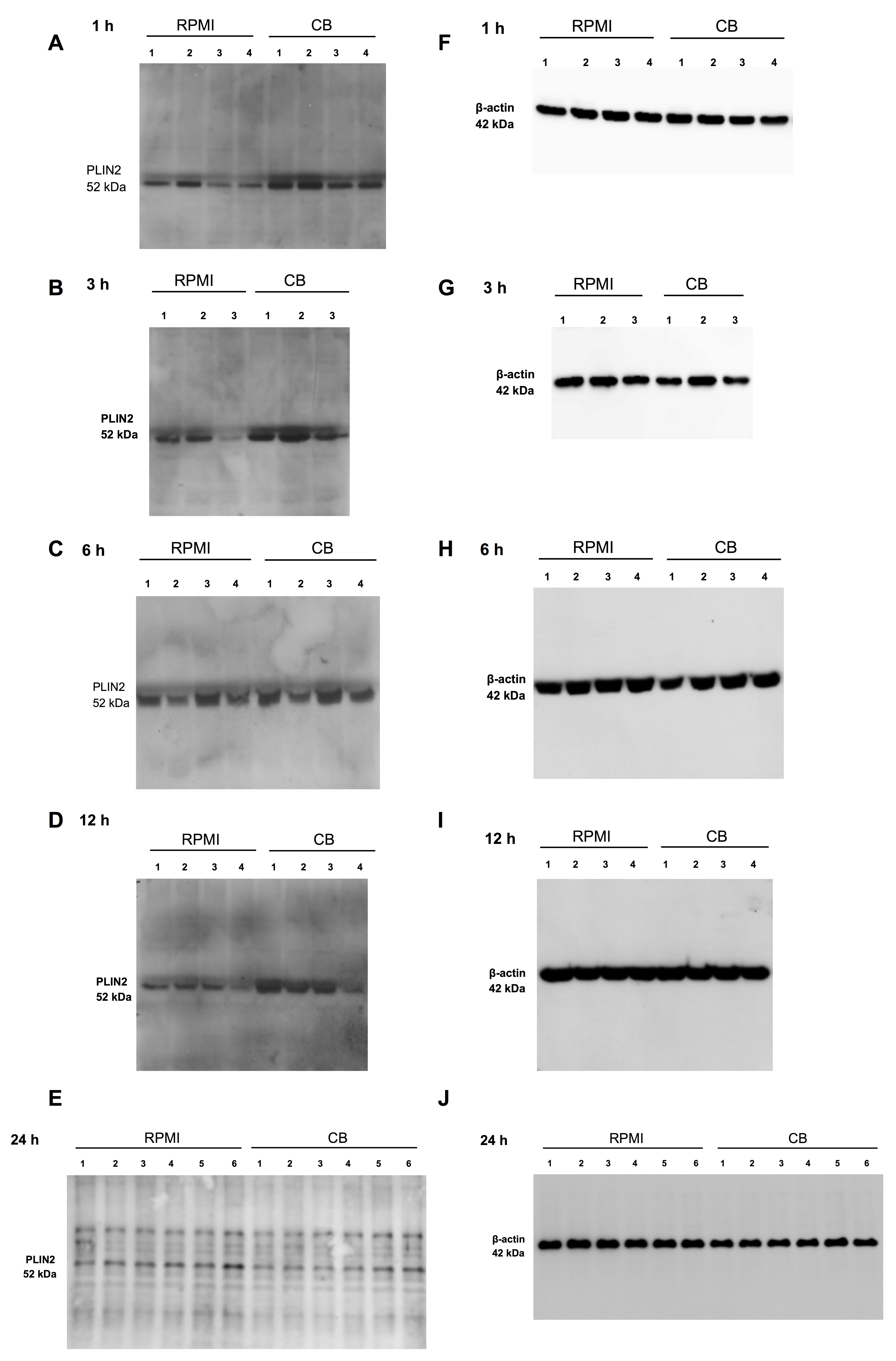


Supplementary Figure 1. **CB upregulates PLIN2 protein expression in macrophages.** Peritoneal macrophages were incubated with CB (0.4 µM) or RPMI (control) for 1, 3, 6, 12 and 24 h. Western blotting of PLIN2 and β-actin (loading control) in macrophage extracts. (A-E) Full length blots of PLIN2 and (F-J) β-actin.
